# Impaired spatial and non-spatial configural learning in patients with hippocampal pathology

**DOI:** 10.1016/j.neuropsychologia.2007.04.007

**Published:** 2007

**Authors:** Dharshan Kumaran, Demis Hassabis, Hugo J. Spiers, Seralynne D. Vann, Faraneh Vargha-Khadem, Eleanor A. Maguire

**Affiliations:** a Wellcome Trust Centre for Neuroimaging, Institute of Neurology, University College London, 12 Queen Square, London WC1N 3BG, UK; b School of Psychology, Cardiff University, Tower Building, Park Place, Cardiff CF10 3AT, UK; c Developmental Cognitive Neuroscience Unit, Institute of Child Health, University College London, Great Ormond Hospital for Children NHS Trust, London WC1N 1EH, UK

**Keywords:** Hippocampus, Memory, Configural, Associative, Reinforcement learning, Amnesia, Spatial, Non-spatial

## Abstract

The hippocampus has been proposed to play a critical role in memory through its unique ability to bind together the disparate elements of an experience. This hypothesis has been widely examined in rodents using a class of tasks known as “configural” or “non-linear”, where outcomes are determined by specific combinations of elements, rather than any single element alone. On the basis of equivocal evidence that hippocampal lesions impair performance on non-spatial configural tasks, it has been proposed that the hippocampus may only be critical for spatial configural learning. Surprisingly few studies in humans have examined the role of the hippocampus in solving configural problems. In particular, no previous study has directly assessed the human hippocampal contribution to non-spatial and spatial configural learning, the focus of the current study. Our results show that patients with primary damage to the hippocampus bilaterally were similarly impaired at configural learning within both spatial and non-spatial domains. Our data also provide evidence that residual configural learning can occur in the presence of significant hippocampal dysfunction. Moreover, evidence obtained from a post-experimental debriefing session suggested that patients acquired declarative knowledge of the underlying task contingencies that corresponded to the best-fit strategy identified by our strategy analysis. In summary, our findings support the notion that the hippocampus plays an important role in both spatial and non-spatial configural learning, and provide insights into the role of the medial temporal lobe (MTL) more generally in incremental reinforcement-driven learning.

## 1 Introduction

The hippocampus is widely accepted to play a critical role in memory (Eichenbaum, 2004; Scoville & Milner, 1957; Squire, Stark, & Clark, 2004). However, no consensus has yet been reached regarding the fundamental mechanisms underpinning this function. An influential theory, originally formulated on the basis of experiments in rodents, proposes that the hippocampus primarily processes spatial information, creating and storing representations of the spatial relationships between places in the environment in a world-centred framework (O’Keefe & Nadel, 1978). Problematic for this view (although see: Burgess, Becker, King, & O’Keefe, 2001; Burgess, Maguire, & O’Keefe, 2002), however, is the wealth of evidence demonstrating that damage to the human hippocampus results in impairments on tasks involving no overt spatial component e.g. recall of word pairs (Eichenbaum, 2004; Squire et al., 2004). It has therefore been argued that the hippocampus plays a more general, domain-independent role in memory, associating or binding together the different elements of an experience (Eichenbaum, 2004; McClelland, McNaughton, & O’Reilly, 1995; O’Reilly & Rudy, 2001). According to this perspective, the neural coding of allocentric space is but one example of the kinds of “configural/conjunctive” (O’Reilly & Rudy, 2001; Sutherland, McDonald, Hill, & Rudy, 1989) or “relational” representations (Cohen & Eichenbaum, 1993; Eichenbaum, 2004) mediated by the hippocampus.

The hypothesis that the hippocampus plays a crucial role in binding together multiple elements of an experience has been widely examined in rodents using a class of tasks known as “configural associative” or “non-linear” (Moses & Ryan, 2006; Rudy & Sutherland, 1995; Sutherland et al., 1989). The key feature in a configural associative task is that no single element (i.e. single shape or position) is sufficient to solve the problem. Instead, subjects must represent the outcomes that result from specific combinations of elements (i.e. “configurals”). Although hippocampal lesions in rats have been reported to produce deficits on some types of non-spatial configural tasks (Alvarado & Rudy, 1995; Rudy & Sutherland, 1989), performance on other non-spatial configural tasks (e.g. Davidson, McKernan, & Jarrard, 1993; Han, Gallagher, & Holland, 1998) seems to be largely spared. On the basis of these inconsistent effects of hippocampal damage on non-spatial configural learning, it has been argued that the hippocampus is primarily involved in *spatial* configural learning (Aggleton & Pearce, 2001; Sanderson, Pearce, Kyd, & Aggleton, 2006). Only one study has directly tested this hypothesis, and provided evidence in favour of it. Rats with hippocampal lesions were able to acquire two non-spatial configural tasks (transverse patterning and biconditional discrimination) at a normal rate, but failed at a configural task where space was integral (a “structural” discrimination) (Sanderson et al., 2006). Surprisingly, few studies in humans have investigated the role of the hippocampus in configural learning. In the one study where neuroimaging confirmed that damage was limited primarily to the hippocampus, patients with amnesia took a significantly greater number of trials, but were finally able to solve a non-spatial configural problem, namely the transverse patterning task (Reed & Squire, 1999). However, to our knowledge, the role of the human hippocampus in the learning of a spatial configural discrimination task has not been previously examined, nor compared directly to its role in non-spatial configural learning.

**Fig. 1:**
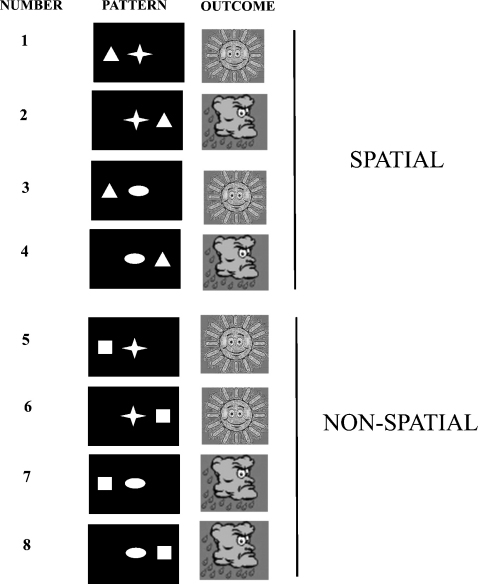
Experimental design: subjects were instructed to play the role of a weather forecaster, and try to learn over the course of the experiment how different “patterns” of shapes on the screen were associated with one of two outcomes, sun or rain (see Section 2). Each one of the eight patterns was associated with an outcome in a deterministic fashion (i.e. with 100% probability). In patterns 1–4, the position of the triangle determines the outcome (in this example, although the allocation of shapes to outcomes was changed between subjects). Hence when the triangle appears on the left, the outcome is sun regardless of the shape present in the centre. When the triangle appears on the right, the outcome is always rain. In patterns 5–8, specific shape–shape pairings determine the outcome, with the position of the square being irrelevant. Hence, square together with star is associated with sun, regardless of the position of the square. Conversely, square together with ellipse is always associated with rain. Trials could therefore be divided conceptually into those involving learning of spatial (patterns 1–4), as opposed to non-spatial (patterns 5–8), configural associative information.

In this study, we explored the role of the human hippocampus in memory for spatial and non-spatial configural associative information in the setting of a novel associative learning task consisting of multiple trials with feedback. Subjects were instructed to play the role of a weather forecaster, and try to learn over the course of the experiment how different patterns of shapes on the screen were associated with one of two outcomes i.e. sun or rain (see Section 2). Each one of eight patterns (Fig. 1) was associated with an outcome in a deterministic fashion (i.e. with 100% probability). As is evident from the upper four patterns (1–4) in Fig. 1 the position of the triangle determines the outcome (in this example, although the allocation of shapes to outcomes was changed between subjects). Therefore, when the triangle appears on the left, the outcome is sun regardless of the shape present in the centre. Conversely, when the triangle appears on the right, the outcome is always rain. From the bottom four patterns (Fig. 1: patterns 5–8), it is evident that it is the specific shape–shape pairings that determine the outcome, with the position of the square being irrelevant. Square together with star is associated with sun, regardless of the position of the square. Conversely, square together with ellipse is always associated with rain. Hence, although all eight patterns were intermixed pseudorandomly throughout the experiment (see Section 2), trials could be divided conceptually into those involving learning of spatial (Fig. 1: patterns 1–4), as opposed to non-spatial (Fig. 1: patterns 5–8), configural associative information. Critically, subjects could only solve the task by learning the outcomes associated with specific shape–location (i.e. spatial configural) and shape–shape (i.e. non-spatial configural) pairings. As such, subjects could not solve the task by using only elemental information (i.e. a single shape or position), and only achieve a maximum of 75% correct responses using this strategy. We also employed a strategy analysis that permitted us to characterize the nature of the information (i.e. elemental versus configural associative) acquired by subjects during learning (see Section 2). Of note, our task, although superficially similar to the “standard” weather prediction task (Poldrack et al., 2001; Hopkins, Myers, Shohamy, Grossman, & Gluck, 2004; Knowlton, Mangels, & Squire, 1996), differs considerably since it involves the learning of configural (as opposed to elemental) information and is deterministic (rather than probabilistic) in nature.

To summarise, our study set out to discover whether amnesic patients with damage to the hippocampus bilaterally would be impaired at an associative learning task that relied upon the acquisition of configural information. In particular, our experimental design allowed us to directly compare the role of the hippocampus in configural learning in the spatial, as opposed to the non-spatial, domain. Moreover, we sought to characterize the performance of patients by using a strategy analysis to determine the nature of the information learnt (i.e. elemental versus configural). Finally, we asked by means of a detailed post-experimental debriefing, whether residual learning capacity in the patients was associated with explicit knowledge of the underlying task contingencies. If so, this would be consistent with residual learning ability being underpinned by areas within the medial temporal lobe (MTL), as opposed to a striatal-based system, given that the MTL is thought to mediate declarative memory (Squire et al., 2004). As such, we hoped to further our understanding of the parameters determining when the MTL predominates over striatal-based systems, even under conditions of hippocampal dysfunction, during incremental reinforcement learning.

## 2 Methods

### 2.1 Participants: patients

**Fig. 2:**
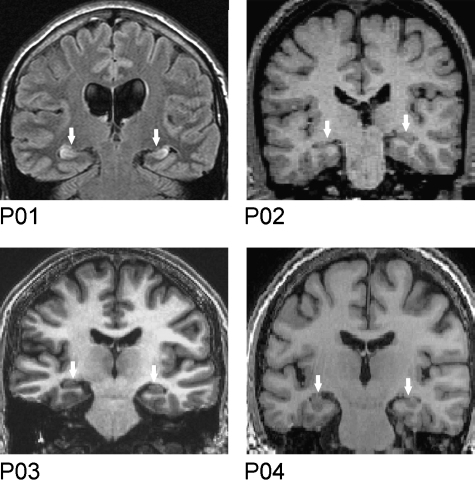
Structural MRI scans: coronal sections through the MRI brain scans of each patient, where the damaged hippocampi are indicated by white arrows. Note P01's scan is a FLAIR image, while the other scans are T1 images.

Four patients took part (all male, one left-handed) each with primary damage to the hippocampi bilaterally, and concomitant amnesia (see below for details of each case). The mean age of the patients was 39 years (S.D. 15.9, range 24–58), years of education 15.5 years (S.D. 3.1, range 12–19) and verbal IQ was 109.5 (S.D. 7.9, range 99–116) (Wechsler, 2001). Lesions were confirmed by structural MRI scans and appeared to implicate the hippocampi, with no evidence of damage in adjacent medial temporal areas (Fig. 2).

#### 2.1.1 Case reports

##### 2.1.1.1 P01

This patient was male, left-handed, and aged 24 at the time of testing. His case has been described elsewhere (Hassabis, Kumaran, Vann, & Maguire, 2007; Samarasekera et al., 2007). To summarise, 3 years earlier he had been at university studying for a PhD. Following a flu-like illness lasting 3 days, he suffered two generalized seizures. He was admitted to the intensive care unit because of persistent seizures that were brought under control after treatment. At that time, he reported symptomatic problems with memory. MRI scan showed high FLAIR signal selectively in both hippocampi (see Fig. 2). The diagnosis was limbic encephalitis. On follow up at 27 months he had been forced to abandon his higher degree because of a persisting memory impairment. In terms of his neuropsychological profile, his verbal IQ was in the high average range (116). At the time of this study, language, perceptual and executive functions were within the normal range. He was impaired on anterograde memory tests in the visual domain in particular, and had a significant temporally ungraded retrograde amnesia for autobiographical events.

##### 2.1.1.2 P02

This patient was male, right-handed, and aged 28 at the time of testing. His case has been described in detail elsewhere (patient Jon: Baddeley, Vargha-Khadem, & Mishkin, 2001; Gadian et al., 2000; King, Burgess, Hartley, Vargha-Khadem, & O’Keefe, 2002; King, Trinkler, Hartley, Vargha-Khadem, & Burgess, 2004; Maguire, Vargha-Khadem, & Mishkin, 2001; Vargha-Khadem et al., 1997). To summarise, this patient suffered perinatal anoxia which resulted in developmental amnesia. There is a volume reduction of approximately 50% in both hippocampi with apparently preserved surrounding cortical tissue (see Fig. 2). Despite this, he attended normal school until aged 18. His memory impairment makes it difficult to work, although he now does so as a gardener's assistant. His verbal IQ was in the average range (108), and his performance normal on language, perceptual and executive tests. His anterograde episodic memory was very impaired, while he appears to be able to acquire and retrieve from long-term memory semantic information (evidenced by normal school attendance and performance on measures of vocabulary, comprehension, semantic judgements and lexical decision; Baddeley et al., 2001). He performed within normal limits on some tests of recognition, but was very impaired on tests of recall. While he appeared to retain memory for some autobiographical events, overall he was significantly impaired (Maguire et al., 2001).

##### 2.1.1.3 P03

This patient was male, right-handed, and aged 47 at the time of testing. His case has been described in detail elsewhere (patient KN: Aggleton et al., 2005; McKenna & Gerhand, 2002). To summarise, this university-educated former industrial biochemist contracted meningeoencephalitis at the age of 34 and then recurrent meningitis. He was left without useful motor function below T12, and amnesia. Furthermore, he has a congruous inferior altitudinal hemianopia consistent with an occipital lobe lesion. Prior to the experiment, we confirmed that the stimuli used were comfortably within P03's field of view. Visual acuity was essentially normal (6/6 right, 6/5 left: corrected). Recent MRI scanning (see Aggleton et al., 2005) showed volume reduction in the hippocampi, reduced by 48.8% in the left (2.88 S.D. below the control mean) and 46.2% in the right (2.86 S.D. below the control mean) (see Fig. 2). In terms of his neuropsychological profile, his IQ was in the high average range (113). He performed normally on tests of language, executive function, and perception (allowing for his visual field deficit). His anterograde memory for episodic information was grossly impaired, while he retained some ability to acquire new semantic information. He performed within normal limits on some tests of recognition, but was very impaired on tests of recall. His retrograde memory for autobiographical events was impaired across four decades.

##### 2.1.1.4 P04

This patient was male, right-handed, and aged 58 at the time of testing, and has been reported previously (Hassabis et al., 2007). He left school aged 11 but later returned to complete a university degree. Prior to his illness he had worked as a market trader for more than 20 years. He presented with sudden onset memory difficulties associated with what he described as ‘panic attacks’ but which were subsequently confirmed as seizures. MRI scan revealed abnormal high signal restricted to the hippocampi and amygdalae. After extensive clinical investigations, it was established that he had limbic encephalitis associated with voltage gated potassium channel antibodies (VGKC-Ab; Vincent et al., 2004). Scanned 18 months later, the abnormalities that were evident on MRI had resolved, although his hippocampi were noted to be small (Fig. 2). He is not able to return to work because of persisting memory problems. In terms of his neuropsychological profile, his verbal IQ was in the normal range (99). Although English was not his first language, limiting the range of tests that could be administered, at the time of this study he performed within normal limits on tests of language, perception and executive function. On anterograde memory tests, his performance was low average–average on tests of verbal and visual recognition. In contrast, his verbal recall was impaired. Testing of retrograde memory revealed amnesia dating back at approximately 10 years for autobiographical events.

#### 2.1.2 Control subjects

Six healthy control participants also took part (all male, one left-handed). The mean age of the control subjects was 43.5 years (S.D. 14.7, range 25–60), years of education was 16 years (S.D. 2.4, range 11–17), and verbal IQ was 107.7 (S.D. 6.6, range 96–114). There was no significant difference between the patients and control subjects on these background characteristics (age *t*(8) = 0.46 *p* = 0.66; education *t*(8) = 0.29 *p* = 0.78; IQ *t*(8) = −0.40 *p* = 0.70). As well as comparing the two groups, we ensured that each patient was closely matched to two of the control subjects on age, education and IQ. The same two control subjects were matched to both P01 and P02. All participants gave informed written consent to participation in the study in accordance with the local research ethics committee.

### 2.2 Experimental design

#### 2.2.1 Tasks and procedures

All testing was performed on a Dell Laptop computer. Subjects entered their responses via two keys marked “S” and “R”, denoting the outcomes “Sun” and “Rain”, respectively. Allocation of keys to outcomes was randomised between subjects.

Stimuli consisted of four simple geometric shapes: square, triangle, star, and ellipse. On each trial, two shapes were displayed, one in the centre of the screen, and one either to the right or the left. As illustrated in Fig. 1, the square and triangle were only presented on either the left or right hand side of the screen, with the star and ellipse only presented in the centre of the screen. There were therefore eight possible “patterns”, reflecting the different combinations in which the shapes could be presented (see Fig. 1). Each shape configuration (e.g. triangle on left, star) was associated with a given outcome (e.g. sun) with a 100% probability. This probability was fixed for the duration of the experiment and did not change. The eight shape configurations are illustrated with their outcomes. Note this is an example since the mapping of shape configurations to outcomes was changed between participants.

Subjects were told to imagine themselves as a weather forecaster who has to predict whether it will be sunny or rainy, on the basis of a given screen which was said to represent constellations of stars in the night sky. Subjects were informed that “it may be the shapes themselves, the location of the shapes or the combination of shapes that predicts the weather”. Subjects were then given a short practice session of eight trials using different shapes from those employed in the main experiment. Task instructions were repeated to subjects three times before the practice session and twice following it. Subjects were told that the reason for the practice was to familiarize them with the task since they would not be able to learn how to predict the weather in this practice session.

Each trial began when two shapes were presented on the screen. Subjects were told to enter their prediction as soon as they were ready. 0.5 s after subjects had responded, the screen advanced to display the outcome for that trial (e.g. picture of sun: Fig. 1), with text informing subjects whether they had made a correct or incorrect response (e.g. “correct”). An auditory tone (duration = 1 s) was also played according to whether subjects had been correct (high-pitched), or incorrect (low-pitched). The screen indicating the trial outcome was displayed for 2 s following which a fixation cross was displayed for 2 s. After this, the next trial began. On a given trial, if subjects failed to respond within 3 s, an instruction appeared “Answer now!”. After a further 2 s, even in the absence of a response, the screen advanced to display the outcome for that trial (e.g. picture of sun), together with text saying “incorrect” and a low-pitched auditory tone. The main experimental session was divided into seven blocks of 50 trials each. Each block lasted approximately 5 min. Each of the eight possible shape configurations was presented pseudorandomly in approximately equal frequencies during each block, with the constraint that no shape configuration was repeated twice in succession. Subjects were given a 1 min break between each block.

If subjects scored 47 or more correct responses within a given block (i.e. criterion of 94%), they were deemed to have solved the task, and the experiment was terminated before the completion of seven blocks. This 94% criterion was chosen to be analogous to the 14/15 criterion (i.e. 93%) used in previous studies of configural learning (Reed & Squire, 1999; Rickard & Grafman, 1998). For the purposes of analysis, subjects who had solved the task prior to completion of the seven blocks were considered to have maintained their final level of performance during each of the remaining blocks, thus equating the quantity of data for patients and control subjects.

#### 2.2.2 Underlying task contingencies

As is evident from the upper four patterns (1–4) in Fig. 1 the position of the triangle determines the outcome. In this example, when the triangle appears on the left, the outcome is sun regardless of the shape present in the centre. Conversely, when the triangle appears on the right, the outcome is always rain. From the bottom four patterns (5–8), it is evident that it is the specific shape–shape pairings that determine the outcome, with the position of the square being irrelevant. Hence square together with star is associated with sun, regardless of the position of the square. Conversely, square together with ellipse is always associated with rain. For the upper four patterns, therefore, the outcome is determined by shape–location conjunctions (i.e. triangle on the left) with shape–shape pairings being irrelevant. Trials when one of these patterns was presented were therefore denoted “spatial”. In contrast, in the lower four patterns, outcomes are determined by specific shape–shape pairings, with shape–location conjunctions being irrelevant. Trials involving these patterns were therefore termed “non-spatial”.

#### 2.2.3 Post-experimental debriefing

Following the completion of the main experimental session, subjects were carefully debriefed in order to evaluate the presence and nature of explicit knowledge concerning the task structure. In this respect, it is worth noting that the task can be successively solved if one acquires *flexible* (or abstracted) knowledge concerning the underlying task structure, for example that it is the position of the triangle that determines the outcome irrespective of the central shape. Alternatively, the task might be solved entirely on the basis of more specific or concrete knowledge, that is the outcome associated with each of the eight patterns, without a more abstract understanding of the task structure. Our debriefing protocol aimed to gain an insight into the extent to which subjects had acquired a flexible, as compared to a specific, knowledge of the task contingencies.

Subjects were first asked an open-ended question requiring them to describe strategies they had used in performing the task, and to state how they thought outcomes were related to shape configurations (i.e. the underlying task structure). Subjects were next asked to estimate outcome probabilities in a given situation. Importantly, subjects were not queried about specific patterns that they had experienced (e.g. square left, ellipse centre) but about more general situations (e.g. square left, regardless of shape in the centre: see below). This was done in order to assess the flexibility of subject's knowledge of the underlying task structure. We do acknowledge, however, that it is possible to arrive at the correct answer to such a question even if one possesses only specific knowledge of the outcome associated with each pattern, by computing average probabilities “on-line”. That patients performed poorly on this test suggests that they were unable to use this strategy (see Section 3).

The questioning was as follows:

“What percentage of the time were the following associated with a sunny outcome? From 0–100. If unsure, please make best guess!”
  Square on the left (regardless of the identity of the shape in the centre)
  Square on the right (“”)
  Triangle on the left (“”)
  Triangle on the right (“”)
What percentage of the time were the following associated with a sunny outcome? From 0–100. If unsure, please make best guess!
  Square and star (regardless of position of the square)
  Square and ellipse (“”)
  Triangle and star (regardless of the position of the triangle)
  Triangle and ellipse (“”).

The test was scored as follows: a subject's estimated probabilities were compared to the correct answers (i.e. true probabilities) for each question. The average deviation that subjects’ answers deviated from the true probabilities was calculated across all the eight questions (i.e. 0% if all answers correct). We reasoned that if a subject was answering randomly (i.e. at chance level), then his/her answer to each question would on average be 50%. The true probabilities for each question are either 0% (e.g. how often is a triangle on the right associated with a sunny outcome?), 50% (e.g. how often is a square on the left associated with a sunny outcome?), or 100% (e.g. how often is a triangle on the left associated with a sunny outcome?). It therefore follows that the score expected by chance in this test is an average deviation of 25% from the true probabilities. In this way, we were able to determine if the average deviation for each subject from the correct probabilities was significantly different to that expected by chance.

Subjects were then shown pictures of each of the eight shape configurations in turn, as they appeared on the screen, and for each configuration were asked: “Was the following “screen” associated with a sunny, or rainy, outcome? If unsure, please guess!”. In contrast to questions requiring subjects to estimate outcome probabilities, this test can be completed successfully on the basis of specific knowledge of the underlying task contingencies (i.e. the outcome associated with each of the eight patterns).

#### 2.2.4 Strategy analysis

We investigated different types of strategy that subjects may have used during the task, using the methodology adopted by Gluck, Shohamy, and Myers (2002), albeit in the context of a task with a different underlying structure. The utility of strategy analyses has recently been highlighted (Gluck et al., 2002) given that the same overall level of performance can arise from the use of different strategies. This is an important issue since the use of different strategies reflects the acquisition of different types of information (e.g. elemental versus configural). In our task, for example, the use of an elemental (i.e. single shape) strategy would result in 75% correct responses if perfectly applied (50% spatial condition, 100% non-spatial condition). Similarly, if a subject used non-spatial configural information in the non-spatial condition, but responded randomly in the spatial condition, an equivalent level of performance would be attained: i.e. 75% overall (50% spatial condition, 100% non-spatial condition). These two different strategies, however, can be distinguished by a strategy analysis. This is the case since each strategy produces a qualitatively different *distribution* of responses across the eight possible shape configurations.

We considered six different types of strategy (see Table 1) ranging from random responding, single cue strategies, to an associative strategy in both spatial and non-spatial trials. For each of these strategies, we used a model to generate the pattern of responses that would be expected if a subject was consistently following this strategy. As in Meeter, Myers, Shohamy, Hopkins, and Gluck (2006), the “assumed consistency parameter” was set at 0.95, defining how reliably a given strategy was followed. For example if a subject was pursuing a single cue strategy, the probability of responding sun when a star was present would be 0.95, and the probability of responding rain would be 0.05.

We focussed on the strategies subjects were using during the last three blocks, or 150 trials, of the experiment since this reflects the subject's final chosen strategy. Indeed, early on, subjects’ responses would be expected to be nearly random with a switch to more optimal strategies occurring with time. Following the procedure in Gluck et al. (2002), we quantified the fit of a subject's individual responses with the responses generated by the model, for each of the six strategies separately. This was done by taking the squared difference between the number of sun responses generated by a subject and the number generated by the model, summed across all eight patterns, for each block. This score was normalised by dividing between the sum of squares of total presentations of each pattern:

$$\text{Score}\;\text{for}\;\text{Model}\;M:\;\frac{\sum_{P}{\left(\#\text{sun}\_\text{expecte}{\text{d}}_{P,M}-\#\text{sun}\_\text{actua}{\text{l}}_{P}\right)}^{2}}{\sum_{P}{\left(\#\text{presentation}{\text{s}}_{P}\right)}^{2}}$$

In which *P* = pattern 1…8; # presentations_*P*_ is the number of times pattern *P* appears in the 50 trials of that block; #sun_expected_*P*,*M*_ is the number of sun responses expected to pattern *P* under model *M*; #sun_actual_*P*_ is the actual number of sun responses the subject made to pattern *P* during that block. Hence the score for Model *M* was a number between 0 and 1 for each strategy. A score of zero would indicate a perfect fit between the model *M* and the subject's response profile across the final three blocks. We compared the scores for each of the six strategies examined and defined the best-fit model as that with the lowest score. If this score was less than 0.1, then this was taken as evidence that the subject was consistently following this strategy during the final three blocks (as in Gluck et al., 2002).

## 3 Results

### 3.1 Statistical analysis

All but one of the control subjects solved the task in an average of 4.4 blocks (S.D. 1.5). The only control subject (matched to P04) who just failed to solve the task, reached 90% correct responses in the final block. None of the four amnesic patients solved (i.e. achieved 47/50 correct responses in a given block: see Section 2) the task within 350 trials (i.e. seven blocks). The overall performance of control subjects, averaged across the entire experiment, was significantly higher than patients (control subjects: 85.8% correct responses (S.D. 8.1); patients: 66.6% (S.D. 6.1); *t*(8) = 4.0 *p* = 0.004).

**Fig. 3:**
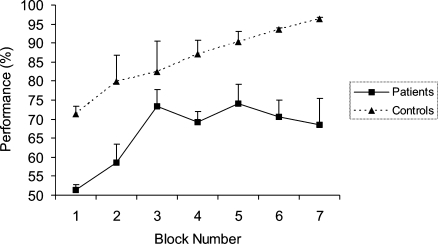
Performance of amnesic patients and control subjects, collapsed across condition (spatial or non-spatial). Each block consisted of 50 trials, with presentation of each of the eight patterns occurring pseudorandomly (see Section 2). Error bars reflect standard error of the mean (S.E.M.).

A repeated measures ANOVA (factors: condition (spatial, non-spatial), block (1–7), group (control, patient)) confirmed a significant between-subjects effect of group (*F*(1,8) = 16.3, *p* = 0.004), with performance of control subjects superior (Fig. 3). There was also a significant effect of block, showing that performance improved over the course of the experiment (*F*(6,48) = 10.8, *p* < 0.001). There was no significant effect of condition (*F*(1,8) = 1.9, *p* = 0.21), no condition–group interaction (*F*(1,8) = 3.2, *p* = 0.11), block–group interaction (*F*(6,48) = 1.4, *p* = 0.26), condition–block interaction (*F*(6,48) = 1.1 *p* = 0.35) or condition–block–group interaction (*F*(6,48) = 0.47, *p* = 0.66).

**Fig. 4:**
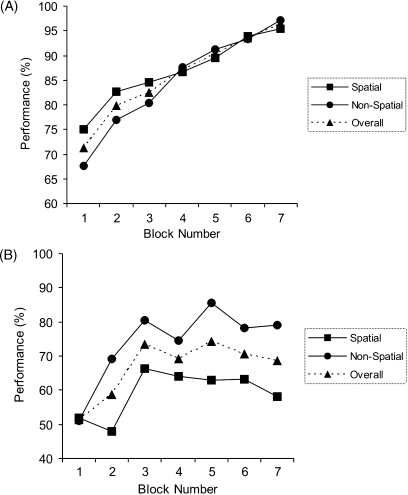
Performance of amnesic patients and control subjects plotted for each condition separately (spatial, non-spatial). (A) Performance of the six control subjects. Error bars are not shown since comparison of performance in spatial and non-spatial conditions is within-subjects. (B) Performance of the four amnesic patients.

We next examined the performance of the two groups separately (Fig. 4) to confirm that there was a significant improvement in patients’ performance over the course of the experiment. When the control subjects were considered alone (Fig. 4A), there was a significant effect of block (*F*(6,30) = 7.2, *p* < 0.001), but no effect of condition (*F*(1,5) = 0.1, *p* = 0.77) and no condition–block interaction (*F*(6,30) = 0.38, *p* = 0.70). This demonstrates that difficulty was well matched between the spatial and non-spatial conditions. With respect to the patients (Fig. 4B), a repeated measures ANOVA (patients alone) confirmed that the patients did indeed learn over the course of the experiment i.e. there was a significant effect of block (*F*(6,48) = 5.7, *p* = 0.002). As expected given the previous results, there was no significant effect of condition (*F*(1,3) = 5.0, *p* = 0.11) or condition–block interaction (*F*(6,18) = 1.0, *p* = 0.46). Although it is apparent from Fig. 4 that the performance of patients was numerically superior in the non-spatial, as compared to the spatial, condition, this difference was not statistically significant (see above). Moreover, subsequent analysis (see below) revealed that this difference was driven by the performance of two “sub-optimal” strategy patients (P02, P03). These patients failed to adopt a configural strategy, instead employing at best an elemental single shape strategy which naturally results in superior performance in the non-spatial condition. Indeed, if perfectly applied, a single shape strategy (i.e. responding according to the identity of the central shape) results in a performance of 100% in the non-spatial condition, and 50% in the spatial condition. Importantly, therefore, the apparent difference between the spatial and non-spatial conditions does *not* relate to differences in spatial and non-spatial *configural* learning, but arises due to use of an elemental strategy by a proportion of the patients.

**Fig. 5:**
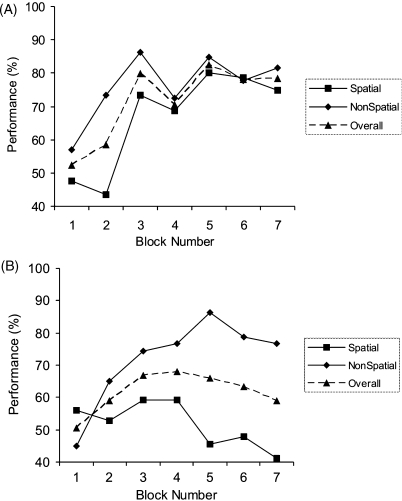
Performance of amnesic patients divided into two groups according to strategy (optimal or sub-optimal). (A) Average performance of optimal strategy patients (P01 and P04). These patients adopted a configural associative strategy (see Section 3/Section 2 for details of strategy analysis) and performed relatively well on the task (average performance during last three blocks: 80%, S.D. 2.9). (B) Average performance of sub-optimal strategy patients (P02 and P03). These patients performed relatively poorly (average performance during last three blocks: 62.7%, S.D. 5.6), failing to adopt a configural strategy and using at best an elemental (i.e. single shape) strategy. The use of this elemental strategy naturally results in superior performance in the non-spatial, as compared to the spatial, condition, although this difference was not statistically significant.

**Fig. 6:**
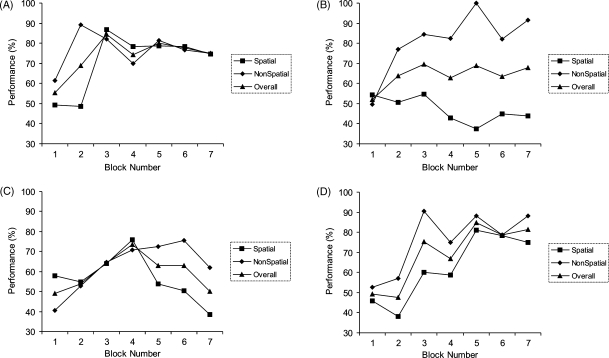
Performance of the amnesic patients with the data for each plotted separately. (A) P01, (B) P02, (C) P03, (D) P04.

We next examined the performance of the four patients in greater detail (see Figs. 5 and 6). This revealed that the patients could be split into two groups according to their overall performance on the task. Moreover, a subsequent strategy analysis (see below) demonstrated that the two patients who significantly improved their overall performance across the experiment had adopted an optimal associative strategy, whereas the other two patients had adopted inferior (e.g. elemental) strategies. A repeated measures ANOVA considering the P01 and P04 as a group (see Figs. 5A, and 6A and D: hereafter termed “optimal strategy patients”—see below for strategy analysis), demonstrated a significant learning effect over the experiment: i.e. significant effect of block (*F*(6,6) = 5.98, *p* = 0.02), with no effect of condition (*F*(1,1) = 6.5, *p* = 0.24) or condition–block interaction (*F*(6,6) = 1.1, *p* = 0.45). As can be appreciated from Fig. 5A, the two optimal strategy patients improved over the first three blocks to a level not significantly different from control subjects (performance in third block: control subjects 82.4% (S.D. 16.1), patients 80% (S.D. 6.4): *t*(6) = 0.25, *p* = 0.81). However, after this point, these two patients failed to improve over the next 200 trials or four blocks: (*r* = 0.15, *p* = 0.8). In contrast, the performance of controls continued to improve (*r* = 0.98, *p* = 0.001).

In contrast, patients P02 and P03 (see Figs. 5B, and 6B and C; hereafter termed “sub-optimal strategy patients”) were not found to exhibit a significant improvement in their performance over the experiment, with no significant effect of block (*F*(6,6) = 2.0, *p* = 0.21). Instead there was a significant condition–block interaction (*F*(6,6) = 7.0, *p* = 0.02) reflecting that they improved their performance in the non-spatial condition, but not the spatial condition, over the seven experimental blocks. As mentioned above, the superior performance in the non-spatial condition observed in these patients arises due to the use of a single shape strategy (i.e. elemental), confirmed by our strategy analysis (see below). It should also be noted that these two patients were found to perform significantly above chance during the experiment: average performance (for both patients, across all blocks, collapsed across condition = 61.8% (S.D. 6.2); *t*(6) = 5.1 *p* = 0.002 one-tailed *t*-test).

### 3.2 Strategy analysis

The strategy analysis followed the procedure outlined in Gluck et al. (2002), and was performed by quantitatively comparing each subject's data with model-generated data reflecting the consistent use of a particular strategy (see Section 2). We considered six different types of strategy (see Table 1) ranging from random responding, single cue strategies, to the optimal associative strategy in both spatial and non-spatial trials. Our list of strategies, therefore, encompasses the main types of possible strategies. We focussed on the last three blocks of the experiment since these reflect the ultimate strategy adopted by a subject.

As expected, the five control subjects who solved the task were best characterized by a spatial and non-spatial associative strategy (i.e. strategy 1). The 1 control subject who did not solve the task was found to adopt a single shape strategy (i.e. strategy 2) during the first four blocks, before later switching to the use of the spatial and non-spatial associative strategy in the final block, during which his performance reached 90%.

Two patients (P01, P04) were found to be fitted by a spatial and non-spatial associative strategy (i.e. strategy 1) during the last three blocks (see Table 2). These patients form the “optimal strategy patients” group (also see Figs. 5A and 6A and D). This finding provides evidence that these two patients acquired configural associative information during the experiment, and that their responses during the last three blocks reflected the use of this knowledge. That these two patients did not solve the task reflects their *imperfect* application of this optimal strategy.

Two patients (P02, P03) formed the “sub-optimal” strategy group (see Table 2) (also see Figs. 5B and 6B and C). P03's data was best-fit by a single shape strategy (i.e. responding according to the identity of the central shape: strategy 2) during the last three blocks. The reason why P03 opted for an elemental single shape strategy rather than an elemental single position strategy (i.e. responding according to if a shape is present or not in the peripheral position) is not clear. It is interesting to note, however, that the only control subject who failed to solve the task employed a single shape strategy during the first few blocks of the experiment, before subsequently adopting the optimal configural strategy. It may be the case that it is the specific nature of our task that favours responding on the basis of a central shape, rather than a peripheral location, if an elemental strategy is adopted. In contrast, P02's data was best-fit by a random strategy (i.e. strategy 6) during the last three blocks (see Table 2). Of note, this does not demonstrate that he was following a random strategy, but merely that his data was not well fit by any of the other strategies, perhaps reflecting switching between different strategies.

Of note, there was no difference between these two groups of patients in terms of age (optimal: 41 years, sub-optimal 37 years), IQ (optimal 107.5, sub-optimal 111.5), or years of education (optimal 15.5 years, sub-optimal 15.5 years: all *p*-values > 0.2). In order to further assess any possible effects of age, we performed a repeated measures ANOVA (factors: block (1–7), group age (young, middle-aged)) where the patients were subdivided into two groups according to their ages (P01, P02: young versus P03, P04 middle-aged). This confirmed that there was no significant between-subjects effect of group age (*F*(1,2) = 0.1, *p* = 0.99), and no significant block × group age interaction (*F*(6,12) = 0.99, *p* = 0.47). Further, when the controls were considered separately, there was no significant correlation between subject age and overall performance, averaged across the entire experiment (*p* = 0.42).

### 3.3 Debriefing

#### 3.3.1 Open-ended questioning

Following the completion of the main experiment, subjects participated in a debriefing session aimed at evaluating the quality of explicit knowledge that subjects had acquired about the underlying task contingencies (see Section 2). All control subjects (including the one subject who just failed to solve the task, reaching only 90% correct responses in the final block) were able to spontaneously produce a succinct description of the underlying task structure, in response to the initial open-ended questioning. This suggests that control subjects had not merely memorized the outcome associated with each pattern, but instead had acquired a flexible or abstracted knowledge of the underlying task contingencies. A representative (abridged) summary of one control subject's description was as follows: “I realised that if triangle on left, this meant sun; if it was on the right, that meant rain; then I realised that square and ellipse meant rain, and square and star meant sun irrespective of position.”

The two “optimal strategy” patients were able to spontaneously describe several, though not all, of the features of the task structure correctly. Interestingly, neither subject was able to describe the underlying task structure in a fully abstract way, as exemplified by the control subjects. The two optimal strategy patients’, P01 and P04, verbal reports were that: “triangle left and ellipse meant sun; triangle right and ellipse meant rain; I think square and ellipse meant rain; but I found star more difficult” (P01), and “combinations of shapes were important; triangle and star was sun; triangle and ellipse was rain; square caused me more problems; think square left star centre was sun.” (P04). P03 reported using a single shape strategy although he was aware that the task could not be solved in this way: “Ellipse meant sun and star meant rain; but I realised it was more complicated”. P02 was aware that the combination of shapes, and their positions, was the key to solving the task, but found the task difficult.

The strategies that patients reported during the debriefing session appeared to correspond to the best-fit strategy identified in our strategy analysis. For example, P03 was fit by a single shape strategy, and reported use of this strategy at debriefing. The optimal strategy patients were both best-fit by a configural strategy and demonstrated explicit knowledge of this information at debriefing.

#### 3.3.2 Probability estimates

Subjects were asked to report the probabilities with which shapes-location, and shape–shape pairings were associated with a sunny outcome (see Section 2). We reasoned that if subjects had merely memorized the outcome associated with each pattern, then they would find this questioning difficult and perform poorly. This is because each question does *not* pertain to a specific pattern (e.g. “how often was a triangle on the left associated with a sunny outcome, regardless of the shape in the centre?”), but rather to a more general situation. As such, this line of questioning taps into the extent to which subjects’ knowledge of the underlying task structure can be considered flexible or abstracted in nature.

Patients did indeed perform poorly on this test, not scoring significantly better than chance (*t*(3) = −0.40, *p* = 0.72) (average deviation from true probability—controls: 4.0% (S.D. 8.5), patients: 23.0% (S.D. 9.5): see Section 2). This was the case even for the two optimal strategy patients who seemed to perform no better than the sub-optimal strategy patients, also close to chance levels. Control subjects, in contrast, were easily able to accurately report the required probabilities without hesitation, scoring significantly above chance: *t*(5) = −6.0, *p* = 0.002. This suggests that controls acquired a flexible knowledge of the underlying task contingencies, whereas patients relied on a more specific knowledge of the outcome associated with each individual pattern in isolation.

#### 3.3.3 Testing of outcomes associated with each pattern

At the end of the debriefing session, subjects were asked to state the outcome associated with each of the eight patterns (see Section 2). This test assesses explicit knowledge of the outcome associated with each pattern. We reasoned that if patients had memorized the outcome of each pattern in isolation, they should perform relatively well in this test (as compared to the probability estimate test). The average performance of the optimal strategy patients was 81% (P01: 6/8; P04: 7/8), and of the sub-optimal strategy patients 69% (P02: 5/8; P03: 6/8) (overall average: 75% (S.D. 10.2)). Of note, the distribution of patients’ responses appeared to be consistent with the best-fit strategy identified, and their spontaneous verbal reports. For example P03 scored 6/8 with his responses consistent with the use of a single shape strategy, which he indeed confirmed verbally. P01 answers, although also yielding a score of 6/8 on this test, appeared to fit better with the use of an imperfectly applied configural strategy. In contrast to their performance on the probability estimate test (see above), patients performed significantly above chance levels (i.e. 4/8 correct) on the pattern-outcome test: *t*(3) = −108, *p* < 0.001. It should be noted that the pattern-outcome test was always at the end of the session, after the probability estimate test. It would therefore seem difficult to argue that the poor performance of patients on the probability test was due to forgetting. Control subjects performed near perfectly when asked to state the outcomes associated with each pattern: average 96% (S.D. 10.0), and significantly better than patients: *t*(8) = 3.2, *p* = 0.01.

## 4 Discussion

In this study, we demonstrate that amnesic patients with primary damage to the hippocampus bilaterally were unable to solve a novel associative learning task whose solution necessitated the acquisition of configural information. Our results show that patients were similarly impaired at configural learning within the spatial and non-spatial domains. Further, our findings demonstrate that residual configural learning can occur in the presence of hippocampal dysfunction. Moreover, we observed that such residual learning was associated with explicit knowledge of the relevant task contingencies in a post-experimental debriefing session. However, patients performed poorly when asked to estimate outcome probabilities in more general situations, suggesting that their knowledge of the task structure was relatively inflexible and concrete in nature. Interestingly, the explicit knowledge demonstrated by patients in the debriefing session appeared to correspond with the best-fit strategy identified by our strategy analysis. These findings therefore suggest that residual learning in patients relies upon regions within the MTL, rather than a striatal-based system often engaged when learning is supervised (i.e. feedback is given) and occurs over numerous trials. In summary, our results support the view that the hippocampus plays an important role in both spatial and non-spatial configural learning, and provide insights into the role of the MTL more generally in incremental reinforcement-driven learning.

Our findings demonstrate that the human hippocampus participates in both spatial and non-spatial configural learning. This contrasts with recent findings that rats with hippocampal lesions were selectively impaired at a spatial, but not a non-spatial, configural task (Sanderson et al., 2006). It is important to note that there are several differences between the two studies that may explain the qualitatively different findings. Firstly, and perhaps most critically, the two studies were carried out in different species (rodents versus humans). It has often been suggested that rodents more readily approach such incremental tasks with a “habit” strategy, whereas humans tend towards a “declarative” or explicit memorization strategy (Bayley, Frascino, & Squire, 2005; Smith & Squire, 2005). Hence, it may be the case that rodents, but not humans, with hippocampal damage are able to acquire non-spatial configural tasks using a habit-based neural system (e.g. the striatum). Secondly, it is widely recognised that patients with damage apparently limited to the hippocampus bilaterally on MRI, may in fact have sustained injury to other brain regions. Hence it is conceivable that rodents with hippocampal damage perform at a higher level on some configural tasks, as compared to humans with apparently selective hippocampal damage, due to the relatively circumscribed nature of the rodent lesions. Finally, it is important to note that the stimulus characteristics differ in a significant respect between the two studies. In our non-spatial configural task, although the spatial location of shapes was irrelevant to the outcome, the two shape cues were *separated in space*, rather than being part of the same “object” as in the biconditional discrimination used by Sanderson et al. This may be important, since according to the relational (although not the configural) theory, the hippocampus is proposed to be critical to the binding together of different objects, rather than different parts of the same object (Cohen & Eichenbaum, 1993).

All four patients tested in this study had damage to the hippocampus bilaterally, resulting in amnesia sufficiently severe to prevent them from functioning independently in daily life. In our experiment, however, the four patients did not form a homogeneous group either in terms of their overall performance, or according to the best-fit strategy identified in our strategy analysis. Two patients (P02, P03) adopted a sub-optimal strategy at best responding according to elemental information (i.e. single shape) and reaching only 63% during the last three blocks. The reason for the poor performance of these two patients is not clear: in particular no difference was apparent between the two groups (optimal and sub-optimal strategy) in terms of age, IQ, or years of education. It may be the case that these patients adopted an inefficient strategy early on in the experiment, and were unable to switch thereafter to a more optimal strategy. Nevertheless, it is interesting to consider their relatively poor performance on our task in relation to previously reported neuropsychological data on these two patients (Aggleton et al., 2005; Maguire et al., 2001; McKenna & Gerhand, 2002; Vargha-Khadem et al., 1997). Prior volumetric analyses have confirmed that their MTL lesions are apparently restricted to the hippocampus bilaterally (Aggleton et al., 2005; Gadian et al., 2000), implying that their poor performance is unlikely to be explained by extra-hippocampal damage. Interestingly, both patients have been shown to be able to learn new semantic information (McKenna & Gerhand, 2002; Vargha-Khadem et al., 1997), and also exhibit hippocampal activation during functional magnetic resonance imaging (Maguire et al., 2001). The performance of P03 on our task would seem to be consistent with previous demonstrations of his impaired performance on tests of recall (Aggleton et al., 2005; Maguire et al., 2001; McKenna & Gerhand, 2002; Vargha-Khadem et al., 1997), and retention of associative aspects of semantic information (McKenna & Gerhand, 2002; Vargha-Khadem et al., 1997). P02, however, has been shown to exhibit preserved performance on some tests of associative recognition (e.g. face–face associations), but not others (e.g. face–voice, object–location associations) (Vargha-Khadem et al., 1997). It may therefore seem surprising that this patient failed to acquire non-spatial configural information even though his inability to learn spatial configural information may be expected based on these prior findings. One possible explanation is that our task, in contrast to previous tasks in which P02 has been shown to exhibit spared performance, involved the discrimination of overlapping stimulus patterns. Indeed, the hippocampus has been suggested to play a critical role in “pattern separation”, a process that may be important for successfully performing some associative/configural tasks (e.g. transitive inference) (McClelland et al., 1995; Moses & Ryan, 2006).

In contrast, two patients (P01, P04) were found to show significant learning over the course of the experiment, reaching a performance level of approximately 80% in the last three blocks. Of note their performance exceeded that obtainable by use of a elemental strategy (i.e. 75%). Our strategy analysis, not used before in previous studies of configural learning, provides a clear demonstration that these patients relied primarily on the use of configural associative information, rather than an elemental strategy. Results from the post-experimental debriefing session provide evidence that these patients also reported explicit knowledge of the underlying task contingencies. Interestingly, their verbal reports and performance on a test where they were required to state the outcome associated with each of the eight patterns, appeared to correspond with the identified best-fit strategy. We suggest, therefore, that regions within the MTL, rather than a system centred on the striatum, supported residual learning in these patients. Hence, one possible explanation for the superior performance of the optimal strategy patients, as compared to the sub-optimal strategy patients, may be that they benefit from a greater level of residual hippocampal function. In contrast, any preserved learning in the sub-optimal strategy patients (i.e. the use of an elemental strategy in P03) may have instead been supported by MTL cortical areas surrounding the hippocampus.

It is also interesting to consider why these two patients failed to solve the task within 350 trials, despite adopting the optimal strategy early on during the experiment. A striking feature that can be appreciated from Fig. 5A, is that these patients performed at a comparable level, not significantly different from control subjects after 150 trials. However, in contrast to control subjects, their performance failed to improve over the last 200 trials (i.e. four blocks). Hence, the patients’ learning curves suggest that they would not have solved the task even if given many more trials, although we cannot exclude this possibility. Our results, therefore, would appear to run counter to recent formulations of the conjunctive theory (Moses & Ryan, 2006; O’Reilly & Norman, 2002; O’Reilly & Rudy, 2001), which regards the neocortex as a slower, but nevertheless powerful, learner of configural associations mediated by neocortical memory representations of similar *quality* to those instantiated by a functioning hippocampus. As such, learning in the presence of hippocampal dysfunction is therefore predicted to proceed to the same level of performance, albeit at a slower rate as compared to normals.

One possible explanation for the failure of the optimal strategy patients to solve the task may be that they were unable to represent the underlying task contingencies in the most efficient or “flexible” manner, due to hippocampal dysfunction. Although our task can be solved by merely memorizing the associated outcome of each single pattern in isolation from one another, this would seem to be a highly inefficient strategy given the presence of multiple patterns with overlapping elements. Data obtained from the debriefing session suggests this might perhaps be the case. Optimal strategy patients performed well when tested on the outcome associated with each pattern in isolation from one another but close to chance when asked to estimate outcome probabilities in a more abstract manner (see Section 3). This suggests that patients were able to at least partially learn the outcomes associated with each specific pattern, but were unable to relate patterns to one another and therefore achieve a more flexible or abstract representation of the task contingencies as a whole. Our results can therefore be viewed to provide some support for a relational view of hippocampal function, which posits that hippocampal damage produces deficits on configural, or indeed any tasks, according to the degree to which their solution requires the use of “flexible” memory representations, which preserve information about individual elements themselves as well as about their relationship to others (Eichenbaum, 2004; Moses & Ryan, 2006).

There has recently been much interest in the relative contributions of two neural systems, the MTL on one hand and a striatal-based system on the other, in mediating incremental reinforcement-driven learning (Foerde, Knowlton, & Poldrack, 2006; Poldrack & Packard, 2003; Yin & Knowlton, 2006). The circumstances under which one system predominates over the other are still largely uncertain, and the focus of much current research. Factors thought to be important include the duration and amount of training, the ease of memorization associations (e.g. deterministic versus probabilistic), and the relative integrity of the two neural systems in the learner (Yin & Knowlton, 2006). As such, it has been widely held that learning in the “standard” weather prediction task occurs outside awareness, is mediated by a striatal-based system, and proceeds essentially normally in patients with MTL amnesia (Knowlton et al., 1996; Yin & Knowlton, 2006; although see Hopkins et al., 2004). In contrast, our task may rely more heavily upon the MTL rather than the striatum since all pattern-outcome associations were deterministic rather than probabilistic. Moreover, all patterns involved the presentation of two shapes, as compared to the variable number of cues displayed on the screen in the standard weather prediction task. Our results therefore, in demonstrating that incremental associative learning even in the presence of hippocampal dysfunction proceeds declaratively, provide insights into the circumstances under which the MTL plays an important role in reinforcement learning.

Despite many years of study, a key question relating to whether the human hippocampus plays an equal role in memory in the spatial and non-spatial domains has not been definitively answered. The present study represents the first direct comparison in humans of the role of the hippocampus in the learning of configural discriminations in the spatial and non-spatial domains. In so doing, we provide evidence that the human hippocampus plays an important role in configural learning within both domains. In the future, it will be important to determine the extent to which pattern separation processes, which permit multiple overlapping memory representations to be kept separate from one another, underlie the hippocampal contribution to configural learning.

## Competing Interests

The authors declare that they have no competing financial interests.

## Figures and Tables

**Table 1:** Definition of the six strategies used in the strategy analysis

Strategy	Pattern							
	1	2	3	4	5	6	7	8
	T_L_S^a^	T_R_S^a^	T_L_E^a^	T_R_E^a^	SQ_L_S^a^	SQ_R_S^a^	SQ_L_E^a^	SQ_R_E^a^
(1) Spatial + non-spatial associative	*π*	1 − *π*	*π*	1 − *π*	*π*	*π*	1 − *π*	1 − *π*
(2) Single shape	*π*	*π*	1 − *π*	1 − *π*	*π*	*π*	1 − *π*	1 − *π*
(3) Single position	*π*	1 − *π*	*π*	1 − *π*	*π*	1 − *π*	*π*	1 − *π*
(4) Non-spatial associative + random	0.5	0.5	0.5	0.5	*π*	*π*	1 − *π*	1 − *π*
(5) Spatial associative + random	*π*	1 − *π*	*π*	1 − *π*	0.5	0.5	0.5	0.5
(6) Random	0.5	0.5	0.5	0.5	0.5	0.5	0.5	0.5

This table illustrates the probability of responding sun to a given pattern, for each of six strategies. *π* reflects the consistency with which a strategy is reliably followed (i.e. the “assumed consistency” parameter and is set at 0.95; (Meeter et al., 2006). “Pattern” refers to each of the eight patterns as in Fig. 1. “Pattern Description”: (T) triangle, (SQ) square, (S) star, (E) ellipse. SQ_L_S denotes the pattern where square is presented on the left, with star in the middle. For example, if pattern 2 is displayed on the screen, then there is a 5% (i.e. 1–0.95 = 0.05) probability that the response generated will be sun, if strategy 1 (i.e. spatial and non-spatial associative) has been adopted. However, if strategy 2 (i.e. single shape) is being followed, then there is a 95% probability that the response to pattern 2 will be sun.

a Pattern description.

**Table 2:** Results of the strategy analysis

Strategy	Patient			
	P01	P02	P03	P04
1. Spatial + non-spatial associative	**0.08**	0.22	0.25	**0.08**
2. Single shape	0.22	0.18	**0.06**	0.19
3. Single position	0.25	0.29	0.43	0.26
4. Non-spatial associative + random	0.11	0.15	0.14	0.11
5. Spatial associative + random	0.11	0.16	0.33	0.13
6. Random	0.15	**0.08**	0.18	0.16

Scores in this table reflect the fit of each patient's individual responses to responses generated by the model, for each of the six strategy types separately. Scores were obtained by taking the squared difference between the number of sun responses generated by a subject and the number generated by the model, summed across all eight patterns. Score were normalised to yield a number between 0 and 1. The lowest of the six scores pertaining to the six strategy types, if less than 0.1, was taken as evidence that the subject was consistently following this strategy (see Section 2).
